# Comparative pathogenicity of BA.2.12, BA.5.2 and XBB.1 with the Delta variant in Syrian hamsters

**DOI:** 10.3389/fmicb.2023.1183763

**Published:** 2023-06-22

**Authors:** Sreelekshmy Mohandas, Anita Shete, Abhimanyu Kumar, Kundan Wakchaure, Vishal Rai, Chandrasekhar Mote, Hitesh Dighe, Prasad Sarkale, Pranita Gawande, Jyoti Yemul, Annasaheb Suryawanshi, Yash Joshi, Pragya D. Yadav

**Affiliations:** ^1^Maximum Containment Facility, ICMR-National Institute of Virology, Pune, Maharashtra, India; ^2^Department of Veterinary Pathology, Krantisinh Nana Patil College of Veterinary Science, Shirwal, Maharashtra, India

**Keywords:** omicron, pathogenicity, XBB.1, BA.5.2, BA.2.12, hamsters

## Abstract

Omicron variant is evolving into numerous sub variants with time and the information on the characteristics of these newly evolving variants are scant. Here we performed a pathogenicity evaluation of Omicron sub variants BA.2.12, BA.5.2 and XBB.1 against the Delta variant in 6–8-week-old Syrian hamster model. Body weight change, viral load in respiratory organs by real time RT-PCR/titration, cytokine mRNA quantification and histopathological evaluation of the lungs were performed. The intranasal infection of the BA.2.12, BA.5.2 and XBB.1 variants in hamster model resulted in body weight loss/reduced weight gain, inflammatory cytokine response and interstitial pneumonia with lesser severity compared to the Delta variant infection. Among the variants studied, BA.2.12 and XBB.1 showed lesser viral shedding through the upper respiratory tract, whereas the BA.5.2 showed comparable viral RNA shedding as that of the Delta variant. The study shows that the Omicron BA.2 sub variants may show difference in disease severity and transmissibility amongst each other whereas the overall disease severity of the Omicron sub variants studied were less compared to the Delta variant. The evolving Omicron sub variants and recombinants should be monitored for their properties.

## 1. Introduction

SARS-CoV-2 Omicron variant emerged in November, 2021 (World Health Organization, 2020). This Variant of Concern (VOC) has evolved to multiple descendant sub-variants with different set of mutations with time (PANGO lineages, n.d.). Among the sub variants, the BA.5/BA.2 and its descendants are prevalent in recent times. Also, recombinant variants of BA.2 like XBB variant is also increasing in countries like India and Singapore (INSACOG BULLETIN, 2022). The characteristics of the variants may differ based on the key mutations they possess. The laboratory animal models like hACE2 mice and Syrian hamsters for SARS-CoV-2 have been used widely to understand the disease severity of the SARS-CoV-2 variants (Chu et al., 2022). The studies on initial Omicron lineage sub-variants like B.1.1.529, BA.1 in these animal models have demonstrated lesser clinical severity (Suzuki et al., 2022; Yuan et al., 2022). BA.2 was found more replicative in human upper respiratory tract epithelium in *in vitro/in vivo* experiments and more pathogenic in hamster model than BA.1 (Chan et al., 2022; Yamasoba et al., 2022). The BA.2 sub variant was gradually replaced by the more transmissible BA.4 and BA.5 sister clades (Tegally et al., 2022). BA.5 sub-variant showed more fitness and enhanced inflammatory response in comparison to the earlier Omicron sub variants (Tamura et al., 2022). There are also contrasting reports on disease severity about this sub-variant (Wolter et al., 2022; Uraki et al., 2023). The observations of disease severity in humans can be biased due to pre-existing immunity due to natural infection or vaccinations. On 20^th^ November, 2022, the World Health Organization has listed the BA.5, BA.2.75, BA.4.6, XBB and BA.2.3.20 as the Omicron sub variants under monitoring (VUM) (World Health Organization, 2020; Yamasoba et al., 2022). All these are BA.2 descendants except XBB which is a recombinant variant. XBB is a recombinant lineage of BJ.1 and BM.1.1.1 (sub-lineages of BA.2). XBB and its many descendant lineages are classified as the VUM’s.The information about the characteristics of the newly evolving variants is scant. Here we have performed the pathogenicity evaluation of the Omicron sub variants BA.2.12, BA.5.2 and XBB.1 against the Delta variant in 6–8-week-old Syrian hamster model in two separate studies and observed that their disease severity is less compared to the Delta variant.

## 2. Materials and methods

### 2.1. Ethical statement

All the experiments were performed with the approval of Institutional Animal Ethics Committee, ICMR-National Institute of Virology (NIV), Pune and as per the Committee for the Control and Supervision of Experiments on Animals (CCSEA) guidelines of the Government of India in the containment facility of ICMR-NIV, Pune. The animals were procured from the CCSEA licensed laboratory animal facility of ICMR-National Institute of Virology, Pune.

### 2.2. Virus

**Figure 1:**
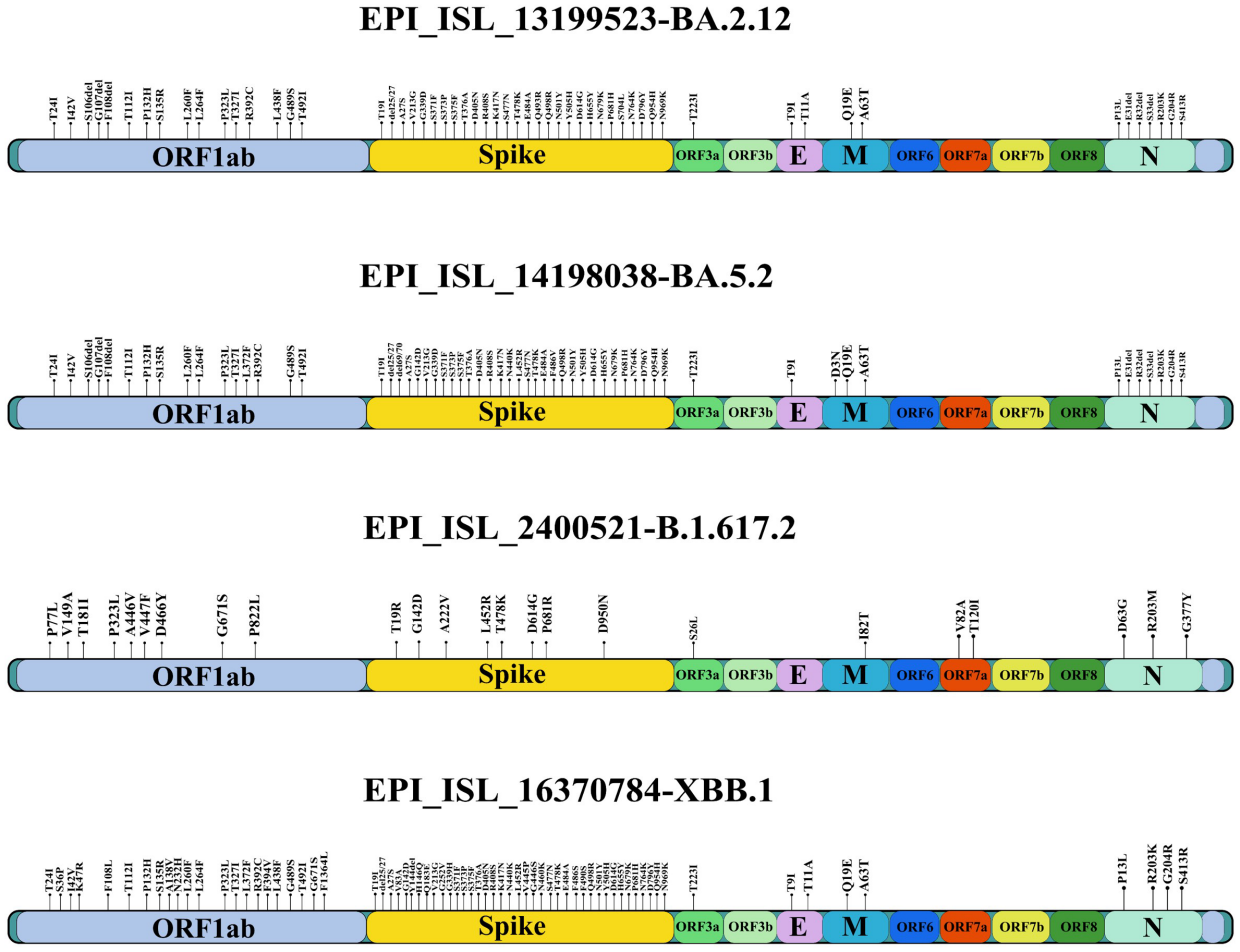
Amino acid substitutions in the SARS-CoV-2 variants used in the study.

The SARS-CoV-2 isolates propagated in Vero-CCL-81 cells (accession numbers are BA.2.12: EPI_ISL_13199523, BA.5.2: EPI_ISL_14198038, B.1.617.2: EPI_ISL_2400521, XBB.1: EPI_ISL_16370784) were used for the study after sequence verification by next generation sequencing (Figure 1). The furin cleavage site of all the isolates were found intact.

### 2.3. Study design

We compared the pathogenicity of the BA.2.12 and BA.5.2 in 6–8-week-old, female, Syrian hamster model with that of the Delta variant (Study 1) and also XBB.1 variant with that of Delta variant in two separate studies (Study 2). In the study 1, a total of 30 animals were infected intranasal with a dose of 3.9 × 10^5^ TCID50 of virus (BA.2.12/BA.5/Delta, n = 10/group, 0.1 mL virus inoculum). The hamsters were monitored for a period of 7 days and nasal wash samples were collected on day 3, 5 and 7 post infection. Five hamsters from each group were euthanized on day 3 and 7 to collect the nasal turbinates and lungs samples. The parameters assessed were body weight loss, viral load in respiratory organs by real time RT-PCR and virus titration; lung histopathology and lung cytokine expression. These criteria were selected based on previous studies demonstrating body weight loss and the respiratory tract infection as the major characteristics of COVID-19 in hamsters (Imai et al., 2020; Mohandas et al., 2020).

We isolated the XBB.1 variant, an Omicron sub variant under monitoring (classified by WHO) by the end of study 1. Hence, we performed a separate study (study 2) to understand its characteristics in comparison to Delta variant. We propagated the virus in Vero-CCL-81 cells and titrated (3.16 × 10^3^ TCID50/0.1 mL) the stock and sequence verified by next generation sequencing. Twenty hamsters were infected with 3.16 × 10^3^ TCID50 of virus (XBB.1/Delta, 0.1 mL virus inoculum, *n* = 10/group). All the parameters as listed for the study 1 were also assessed for this second study. Four hamsters (n = 4/ study) were kept as uninfected control for each study. The body weight changes in the uninfected control group were monitored during the study period. These animals were euthanized at the end of the study period and their lung samples were collected and used for the quantification of cytokine as well as for histopathological evaluation.

### 2.4. Quantitative real time RT-PCR for SARS-CoV-2

The RNA extraction of the swab/wash samples and tissue samples homogenate were performed by MagMAX^™^ viral/pathogen nucleic acid isolation kit (ThermoScientific, United States). Real time RT-PCR was performed for the SARS-CoV-2 genomic RNA (primers targeting E gene) and sub genomic (sg) RNA (primers targeting E gene) using the methods described earlier (Choudhary et al., 2020; Moreira et al., 2021).

### 2.5. Virus titration

As the real time RT-PCR method detects both viable and the non-viable viral particles, virus titration was performed in Vero-CCL-81 cells using the endpoint titration method to understand the live virus load. The nasal turbinate and lungs sample homogenates were titrated for the live virus in Vero (ATCC® CCL-81™) cells (ATCC, United States). The samples in ten-fold dilutions in the media were added onto cells in a 24 well plate. The plate was incubated for one hour. The media was removed after the incubation and the cells were washed with phosphate buffered saline. The maintenance cell culture media containing serum was added onto the cells and was kept for incubation in a CO_2_ incubator for 5 days. The cytopathic effects (CPE) were monitored and the titers were determined by Reed and Muench method.

### 2.6. Histopathological evaluation

For histopathological examination, the lungs samples collected during necropsy were fixed in 10% neutral buffered formalin for a week and were processed during the standard techniques for histopathology (Luna, 1968). The sections were stained by Hematoxylin and eosin. The lung sections were graded to assess the severity of lesions with a score of 1 to 4 for vascular (congestion, haemorrhages, perivascular inflammation), bronchiolar (epithelial loss, degeneration), alveolar architectural changes (emphysema, thickening of septa, pneumocyte hyperplasia, inflammatory cellular infiltration) as well as inflammatory changes like cellular infiltration, exudation and hyaline changes. The cumulative score was compared among different groups.

### 2.7. Relative quantification of cytokine expression

For cytokine/chemokine mRNA quantification, lung samples homogenates were used. The RNA extraction was performed by MagMAX^™^ viral/pathogen nucleic acid isolation kit (Thermo Scientific, United States). The RNA concentrations of the samples were checked in a nanodrop (Nanodrop Technologies, ND 1000) and the RNA concentrations of the samples were adjusted. The lungs samples from the uninfected control animals were used for comparison and the internal control used was HPRT gene. The published primers of IL-1, IL-4, IL-6, IL-10, IL-12 and IFN-γ were used (Zivcec et al., 2011). Delta–delta Ct method was used to estimate the gene expression in fold change.

### 2.8. Statistical analysis

The statistical analysis was performed using GraphPad Prism version 9.1.0 software. The statistical significance was assessed using two-tailed Mann–Whitney test between the groups and the *p*-values less than 0.05 were considered statistically significant.

## 3. Results

### 3.1. Pathogenicity of omicron sub variants BA.2.12 and BA.5.2

**Figure 2:**
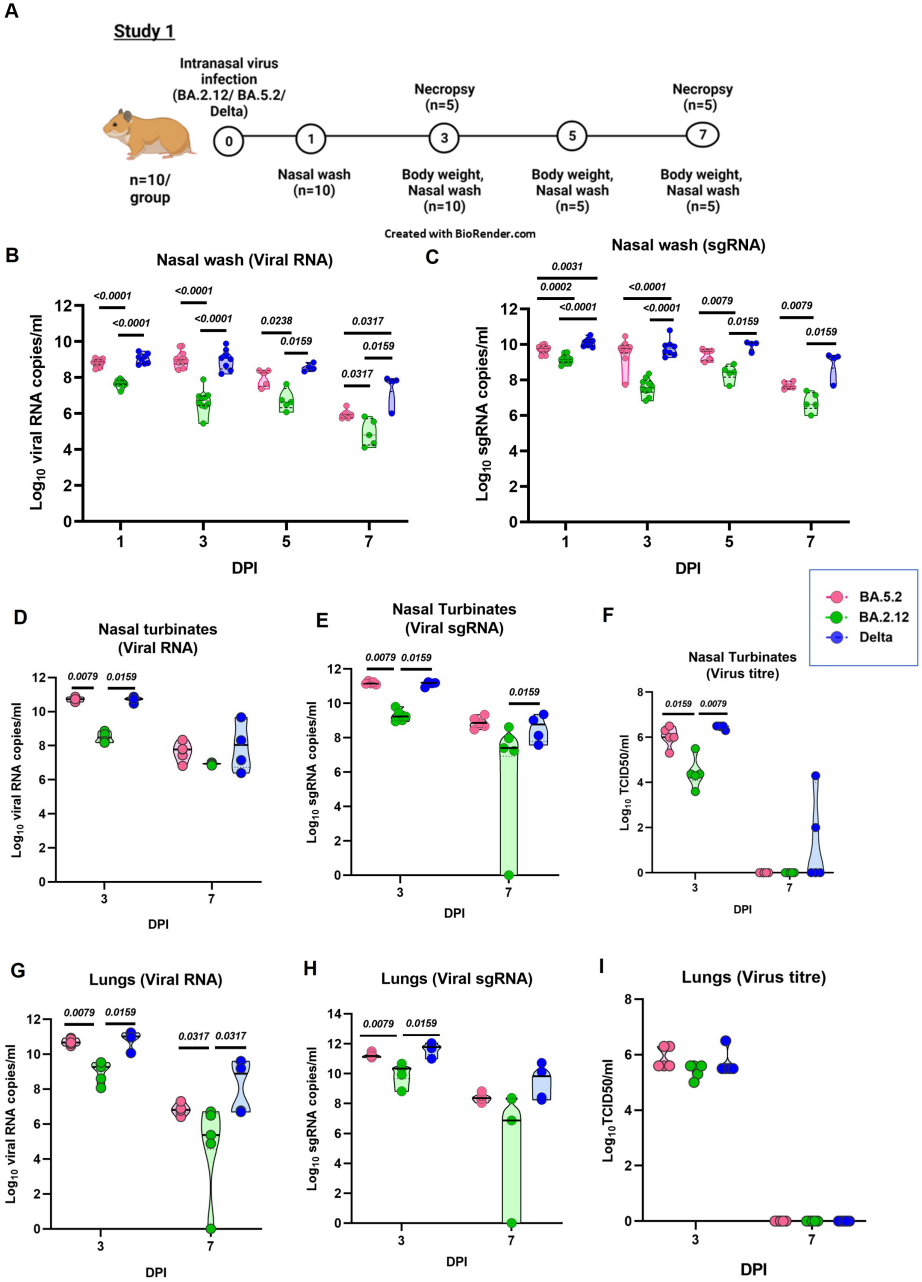
Pathogenicity study of Omicron sub variants BA.2.12 and BA.5.2 in comparison to the Delta variant in the hamster model and the viral load in the respiratory tract. **(A)** Study design. **(B)** Viral RNA and **(C)** viral sub genomic RNA levels in the nasal wash samples collected on day 1 (n = 10/group), 3 (*n* = 10/group), 5 (*n* = 5/group) and 7 (*n* = 5/group) after infection in the study groups. **(D)**Viral RNA, **(E)** viral sub genomic RNA and **(F)** live virus titer in the nasal turbinate samples collected on day 3 and 7 after infection, *n* = 5/group. **(G)** Viral RNA, **(H)** viral sub genomic RNA and **(I)** live virus titer in the lung’s samples collected on day 3 and 7 after infection, *n* = 5/group. Individual values along with the median is plotted in each graph. Mann–Whitney test was used for comparison and the *p* values less than 0.05 were considered statistically significant.

In the study 1, hamsters (n = 10/group) were infected with the either BA.2.12, BA.5.2 and Delta variant (Figure 2A). When the nasal wash viral RNA was compared among different infected groups, BA.2.12 infected groups showed significantly lower viral RNA/sg RNA compared to the BA.5.2 and Delta infected hamsters, whereas BA.5.2 infected hamsters showed similar levels as that of Delta infected animals (Figures 2B,C). Nasal turbinate viral load was also significantly lower in the BA.2.12 compared to BA.5.2 and Delta (Figures 2D–F). Lungs viral titers were also lower in the BA.2.12 infected group (Figures 2G–I).

**Figure 3:**
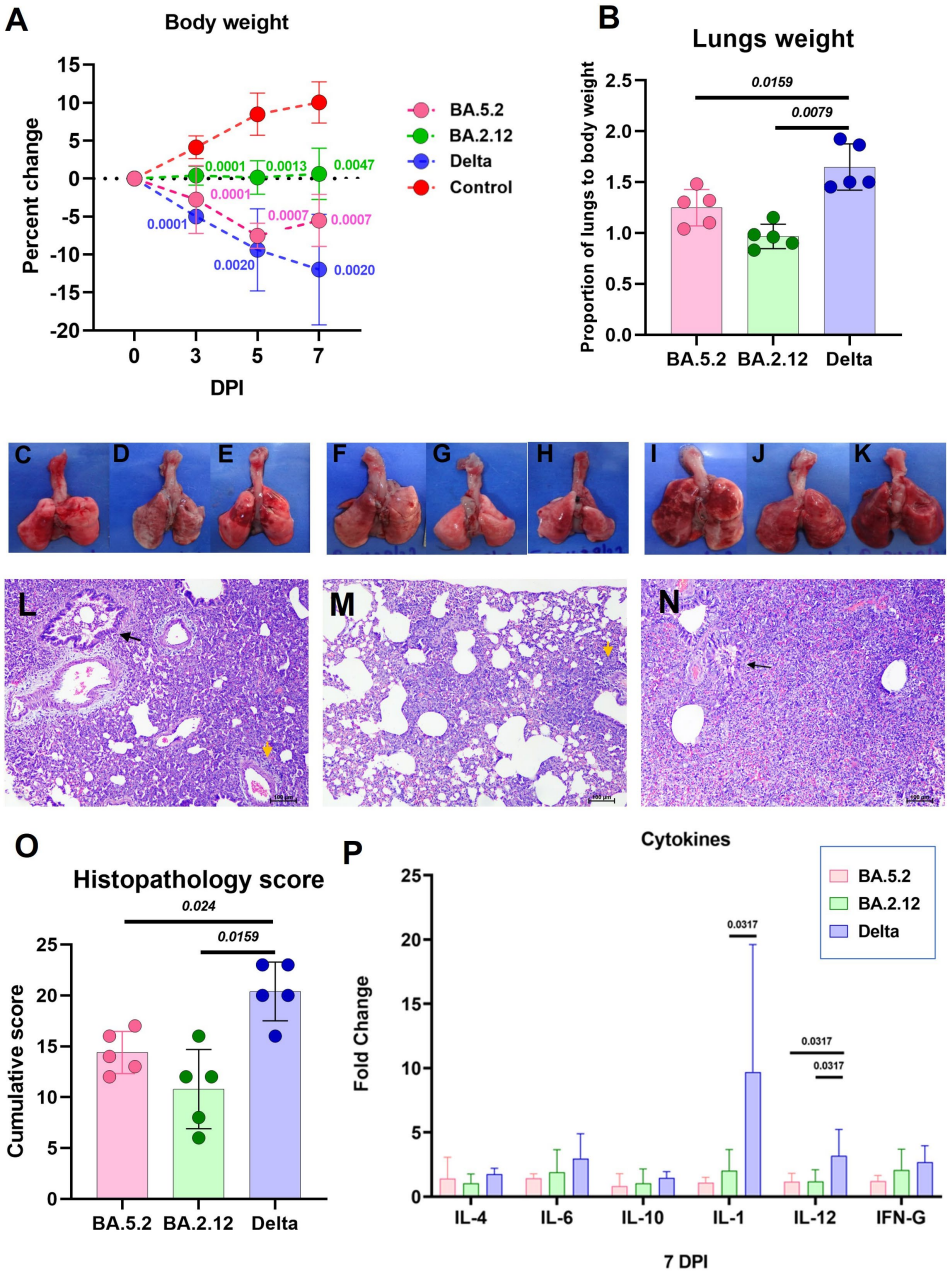
Disease severity in hamsters infected with the Omicron sub variants BA.2.12 and BA.5.2 in comparison to the Delta variant. **(A)** Body weight change in hamsters after infection on day 3 (*n* = 10/ group), 5 (*n* = 5/ group) and 7 (*n* = 5/ group). Mean along with the standard deviation is plotted on the graph. **(B)** Proportion of lungs to body weight in the hamsters infected on day 7 after infection, *n* = 5 /group. Mean along with standard deviation is plotted on the graph. Lungs of hamsters infected with the BA.5.2 **(C–E)**, BA.2.12 **(F–H)** and Delta variant **(I–K)** showing congestion and hemorrhages. **(L)** Lung section of hamster infected with BA.5.2 showing broncho-interstitial pneumonia (black arrow: bronchiole filled with inflammatory exudate, yellow arrow: perivascular inflammatory cell infiltration), H&E, 100 μm on day 7 after infection. **(M)** Lung section of hamster infected with BA.2.12 showing interstitial pneumonia (yellow arrow: perivascular inflammatory cell infiltration), H&E, 100 μm on day 7 after infection. **(N)** Lung section of hamster infected with Delta variant showing broncho-interstitial pneumonia (black arrow: bronchiole filled with inflammatory exudate) with alveolar haemorrhages, H&E, 100 μm on day 7 after infection. **(O)** Cumulative lung histopathology score of the hamsters of 7 days post infection represented as individual animal score along with mean and standard deviation, *n* = 5/ group. **(P)** Cytokine mRNA expression in the lung samples expressed as mean along with the standard deviation, *n* = 5/ group. Mann–Whitney test was used for comparison and the p values less than 0.05 were considered statistically significant.

The body weight loss was highest in the Delta variant [mean ± Standard Deviation (SD) = −11.9 ± 7.27 on day 7] infected animals followed by the BA.5.2 (mean ± SD = −5.5 ± 3.46) and BA.2.12 (mean ± SD = 0.62 ± 3.38) variant (Figure 3A). The lungs body weight ratio was significantly lower in the BA.2.12 and BA.5.2 infected groups (Figure 3B). Grossly, lungs showed patchy distribution of hemorrhages in BA.5.2 infected hamsters (Figures 3C–E), whereas the only few hemorrhagic foci were observed with BA.2.12 infection (Figures 3F–H). In case of Delta variant infection hemorrhages were diffuse (Figures 3I–K). Mild congestion, hemorrhages and bronchiolar epithelial loss were observed, whereas inflammatory changes were severe like alveolar exudation, cellular infiltration in the alveolar septa and peribronchial/ perivascular region were observed in hamsters on day 7 post infection. Occasionally, bronchiolar exudation with inflammatory infiltrates were also observed (Figures 3L–N). The cumulative score for these changes were significantly lower in the BA.5.2 and BA.2.12 infected hamsters (Figure 3O). Altogether, the lung disease severity in the hamsters was less in BA.2.12 and BA.5.2 compared to Delta variant. The cytokine mRNA expression showed slight upregulation of cytokines like IL-1, IL-6, IL-10, IL-12 and IFN- γ in Delta variant infected hamsters compared to the other groups. IL-1 and IL-12 were significantly upregulated in the Delta variant infection (Figure 3P).

### 3.2. Pathogenicity of XBB.1 in Syrian hamsters

**Figure 4:**
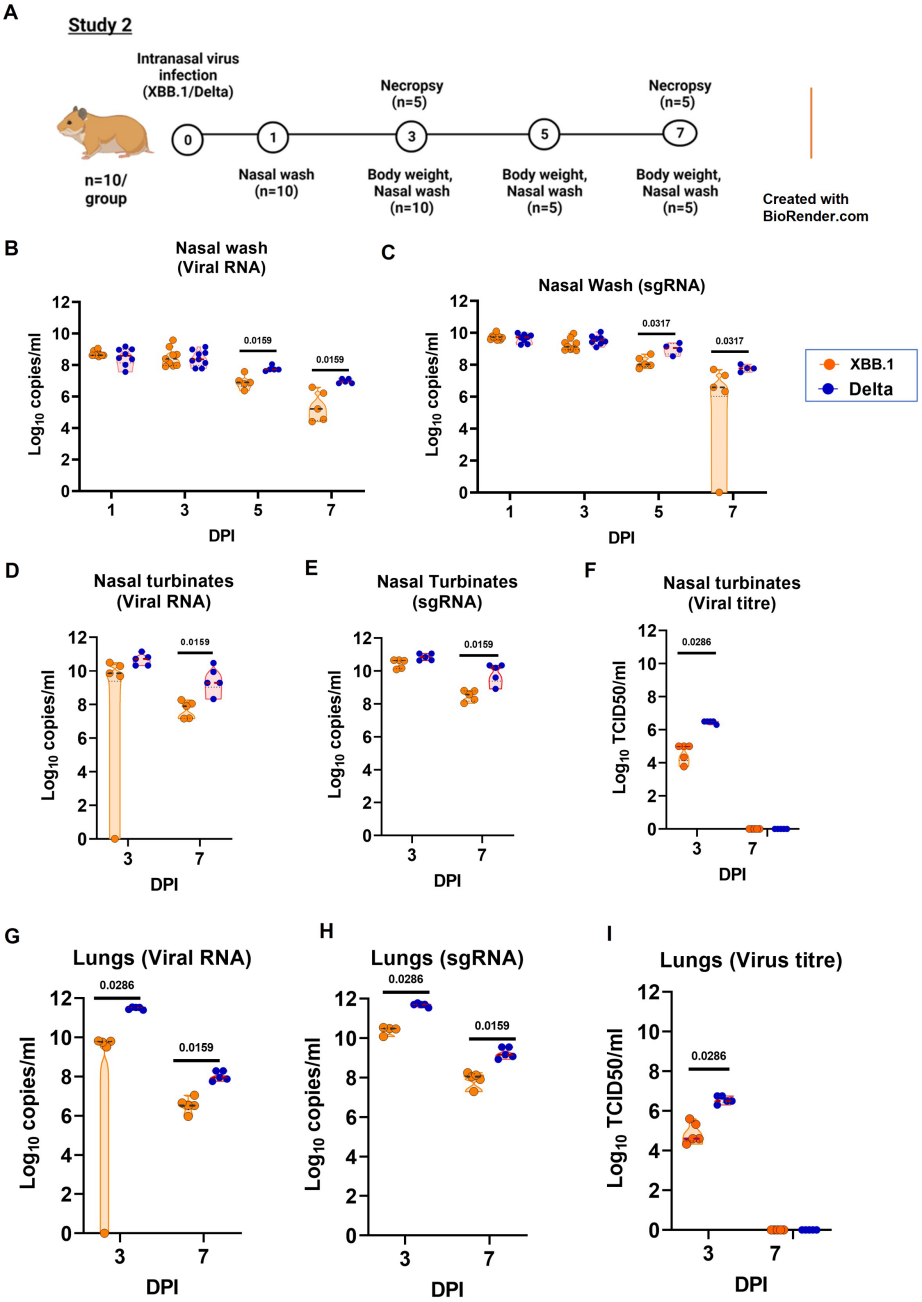
Pathogenicity study of XBB.1 in comparison to the Delta variant in the hamster model and the viral load in the respiratory tract. **(A)** Study design. **(B)** Viral RNA and **(C)** viral sub genomic RNA levels in the nasal wash samples collected on day 1 (*n* = 10/group), 3 (*n* = 10/group), 5 (*n* = 5/group) and 7 (*n* = 5/group) after infection in the study groups. **(D)**Viral RNA, **(E)** viral sub genomic RNA and **(F)** live virus titer in the nasal turbinate samples collected on day 3 and 7 after infection, *n* = 5/group. **(G)** Viral RNA, **(H)** viral sub genomic RNA and **(I)** live virus titer in the lung’s samples collected on day 3 and 7 after infection, *n* = 5/group. Individual values along with the median is plotted in each graph. Mann–Whitney test was used for comparison and the *p* values less than 0.05 were considered statistically significant.

In the study 2, we compared the disease severity of XBB.1 and Delta variant in Syrian hamsters (Figure 4A). The hamsters (*n* = 10/group) were infected intranasally with a dose of 3.16 ×10^3^ TCID50/ml of virus and were monitored and assessed as mentioned for study 1. The viral RNA level in the upper respiratory tract samples were comparable on initial days ie., day 1 and 3, but were significantly reduced in the XBB.1 group on day 5 and 7 (Figures 4B–E). The nasal turbinate virus load was significantly lower in the XBB.1 group on day 3 (Figure 4F). The lungs samples showed lower viral RNA as well as live virus titers on day 3 and 7 in the XBB.1 infected group (Figures 4G–I).

**Figure 5:**
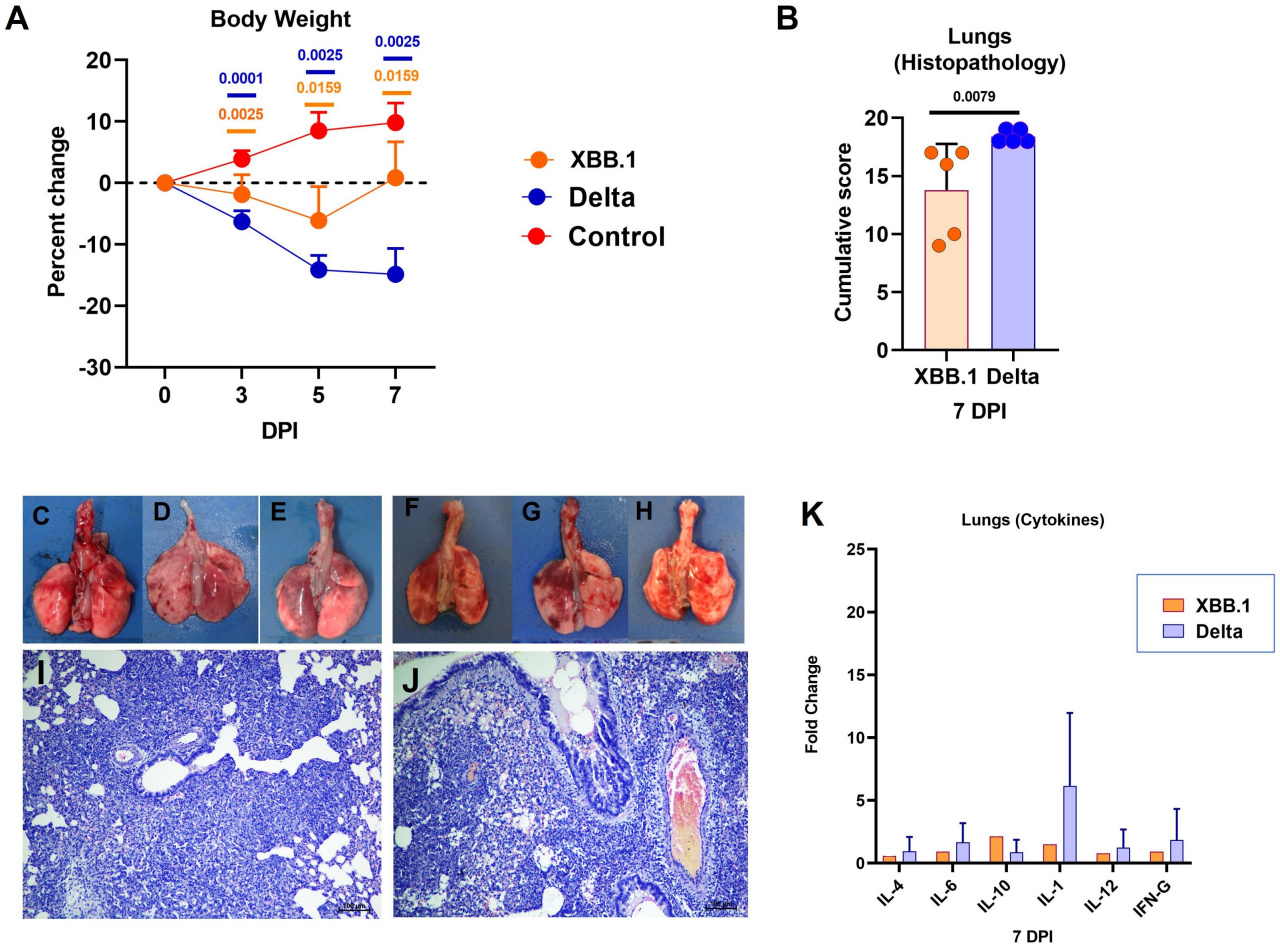
Disease severity in hamsters infected with the XBB.1 variant in comparison to the Delta variant. **(A)** Body weight change in hamsters after infection on day 3 (*n* = 10/ group), 5 (*n* = 5/ group) and 7 (*n* = 5/ group). Mean along with the standard deviation is plotted on the graph. **(B)** Cumulative lung histopathology score of the hamsters on 7  days post infection represented as individual animal score along with mean and standard deviation, *n* = 5/ group. Lungs of hamsters infected with the XBB.1 **(C–E)** and Delta variant **(F–H)** showing congestion and hemorrhages. Lung section of hamster infected with **(I)** XBB.1 and **(J)** Delta variant showing interstitial pneumonia, H& E, 100 μm on day 7 after infection. **(K)** as Cytokine mRNA expression in the lung samples plotted as mean along with the standard deviation, *n* = 5/ group. Mann–Whitney test was used for comparison and the p values less than 0.05 were considered statistically significant.

As observed in the first study, the Delta variant infection caused more weight loss (mean ± SD = −14.8 ± 4.2 on day 7) in comparison to XBB.1 (mean ± SD = 0.85 ± 5.85) (Figure 5A). Broncho-interstitial pneumonia developed in both XBB.1 and Delta variant infected hamsters, whereas the severity by scoring was greater in the latter infected group (Figure 5B). Focal to diffuse hemorrhages were observed grossly in lung lobes of the hamsters of both groups (Figures 5C–H). Engorged blood vessels, perivascular cuffing, diffuse alveolar cellular infiltration, thickening and pneumocytic hyperplasia were observed in both groups. Bronchiolar epithelial loss and inflammatory exudates were observed occasionally (Figures 5I,J). Slight upregulation of the cytokines was observed in the lung samples by relative quantification, but were not statistically significant (Figure 5K).

## 4. Discussion

Based on various studies and the human clinical severity data of Omicron sub lineages, current prevalent lineages like BA.5, BQ.1, XBB, BA.2.30, BA.4.6 does not show any indication of increased disease severity (World Health Organization, 2022). Laboratory animal models like Syrian hamsters have been instrumental to evaluate properties of different SARS-CoV-2 variants and efficacy of many therapeutics and vaccines (Chu et al., 2022). Syrian hamster model mimics the COVID-19 disease characterized by upper respiratory tract infection, body weight loss and development of broncho interstitial pneumonia (Imai et al., 2020). The studies of the earlier VOC’s in hamster model has helped to demonstrate their disease severity and transmissibility (Mohandas et al., 2021a,b; Cochin et al., 2022; Port et al., 2022). Majority studies in laboratory animal models have demonstrated lesser disease severity of Omicron sub-variants (Suzuki et al., 2022; Yuan et al., 2022).

The increased replication of the virus in the upper respiratory tract can lead to more virus shedding and thus contribute to the transmissibility. Here we observed a significantly lower viral RNA shedding in BA.2.12 infected hamsters than BA.5.2 variant. BA.5.2 and XBB.1 showed similar upper respiratory tract viral load as that of Delta variant. The key mutations in the spike protein of the Delta variant which are attributed to enhance transmissibility like D614G, L452R, L478K are present in these variants (Dhawan et al., 2022). BA.2 sub-variant was shown to have increased transmissibility and growth rates compared to BA.1 (Lyngse et al., 2022). This notion was also supported by some studies which showed that BA.2 variant outcompeted BA.1 to become the dominant variant in the upper respiratory tract when an individual has co-infection with both the sub variants (Gjorgjievska et al., 2022). Chan et al. have shown that BA.2 replicates more efficiently than BA.1 in the nasal turbinate’s of K18-hACE2 mice as a reason for its enhanced transmissibility (Chan et al., 2022). The virological characteristics can differ among the Omicron descendant lineages as BA.2.75 was reported to be more pathogenic and transmissible than the parent BA.2 lineage (Saito et al., 2022).

The body weight loss and severity of the lung pathological changes observed in the present study with the Omicron sub-variants ie., BA.2.12, BA.5.2 and XBB.1 were lesser compared to the Delta variant infected hamsters. Instead of P681R attributed to Delta variant pathogenicity, P681H substitution is present in all Omicron sub variants studied here (Saito et al., 2022). The mutations L452R and T478K attributed to disease severity are present (Dhawan et al., 2022). The role of large number of mutations in the structural and nonstructural proteins other than spike protein in the pathogenesis are still not known. A recent study in transgenic mice and hamster model have demonstrated the lower pathogenicity of BA.2, BA.4 and BA.5 when compared to Delta variant (Uraki et al., 2022). Inflammatory cytokine storm has been demonstrated in severe COVID-19 infection in humans (Tang et al., 2020). Although we could not observe such a hyperinflammatory state here, the levels in Omicron variant infected animals were lesser compared to Delta. SARS-CoV-2 produces a mild to moderate disease in hamsters unlike the severe disease reported in humans and the animals recover from the disease in 2 weeks (Imai et al., 2020; Mohandas et al., 2020). Host immune response studies in hamsters also reported absence of the exacerbated immune response and resulting severe COVID-19 (Castellan et al., 2023). Kimura et al. (2022) has shown that BA.4/BA.5 is more pathogenic compared to BA.2, indicating that properties of Omicron sub variants can vary from the parent BA.2 lineage. In another study, the pathogenicity of XBB in hamsters were found comparable to BA.2.75 variant and less severe compared to Delta variant similar to our observations (Tamura, n.d.). These observations shows that these newly evolved Omicron BA.2 sub clades may show difference in disease severity and transmissibility amongst each other whereas the overall severity is less compared to the Delta variant.

## Data availability statement

The original contributions presented in the study are included in the article/supplementary material, further inquiries can be directed to the corresponding author.

## Ethics statement

The animal study was reviewed and approved by Institutional Animal Ethics Committee, ICMR-National Institute of Virology, Pune.

## Author contributions

PY and SM designed the experiments, performed data analysis, interpretation and writing. SM, AK, and KW performed the animal experiments and data collection. AiS monitored the laboratory sample analysis and interpretation. CM performed the histopathological evaluation. VR, HD, PS, PG, JY, AnS, and YJ contributed to laboratory experiments, data collection and interpretation.

## Funding

The study was supported by the intramural funding of Indian Council of Medical Research to the ICMR-National Institute of Virology, Pune.

## Conflict of interest

The authors declare that the research was conducted in the absence of any commercial or financial relationships that could be construed as a potential conflict of interest.

## Publisher’s note

All claims expressed in this article are solely those of the authors and do not necessarily represent those of their affiliated organizations, or those of the publisher, the editors and the reviewers. Any product that may be evaluated in this article, or claim that may be made by its manufacturer, is not guaranteed or endorsed by the publisher.
